# Supplementary material for: Hypothalamic CNTF volume transmission shapes cortical noradrenergic excitability upon acute stress
Source: EMBO J. 2018 Sep 12;37(21):e100087. doi: 10.15252/embj.2018100087 (PMC6213283; doi:10.15252/embj.2018100087)
Supplement: Supplementary file 1 — Appendix [file EMBJ-37-e100087-s001.pdf]

Annex (Supplementary Material) to:

Hypothalamic CNTF volume transmission shapes cortical noradrenergic excitability upon acute stress (A. Alpár *et al*., The EMBO Journal)

Table of Contents

**Appendix Figure S1:**	Acute stress induces CRH accumulation in projections to hypothalamic ependymal cells lining the 3rd ventricle and the release of ciliary neurotrophic factor (CNTF) into the cerebrospinal fluid.
**Appendix Figure S2:**	CNTFRs in LC and experimental models.
**Appendix Table S1:**	Demography and use of human subjects.

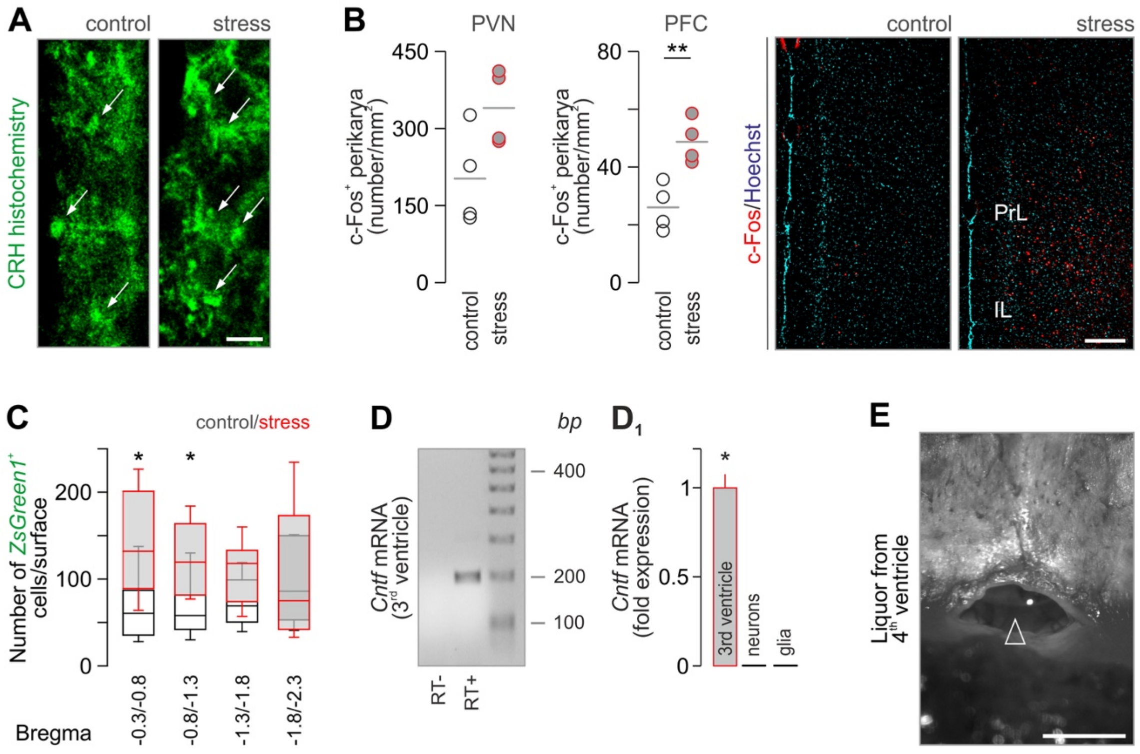

Appendix Figure S1: Acute stress induces CRH accumulation in projections to hypothalamic ependymal cells lining the 3rd ventricle and the release of ciliary neurotrophic factor (CNTF) into the cerebrospinal fluid. (related to Figure 2)

A. CRH+ boutons along the wall of the 3rd ventricle at rest and 20 min after acute stress. Arrows point to CRH-filled bouton-like structures. *Scale bar* = 6 μm.

B. Quantitative histochemistry of c-Fos immunoreactivity in the PVN and PFC 2h after acute formalin stress (n = 4 animals/group). **$p < 0.01$ (Student's t-test). *Abbreviations:* IL, infralimbic cortex; PrL, prelimbic cortex. *Scale bar* = 250 μm.

C. The density of *ZsGreen1*+ terminals in the ependymal layer of the cranial but not caudal 3rd ventricle was increased by acute formalin stress. *$p < 0.05$ (Student's t-test). Box plots represent medians and 10th, 25th, 75th and 90th percentiles from $n \geq$. 3 mice/group. Numerical intervals along the x-axis refer to distance from bregma in mouse (in mm).

D. PCR amplification of *Cntf* mRNA transcripts from microdissected tissues containing cells proximal to the 3rd ventricle in mouse. Amplicons were run on a 1% agarose gel. Samples without reverse transcriptase (RT-) were used as control. (**D**$_1$) *Cntf* expression is likely specific to ependymal cells since neither neurons nor glia, cultured from the rat brainstem (cranial pons) also containing the locus coeruleus (LC) on postnatal day 5, produced *Cntf* mRNA transcripts at levels detectable by real-time PCR. *$p < 0.05$ (Student's t-test) from triplicate experiments. Data were expressed as means ± s.e.m.

E. Cerebrospinal fluid (CSF) was collected from the 4th ventricle, approached through the cerebellomedullary cistern (*open arrowhead*). *Scale bar* = 2 mm.

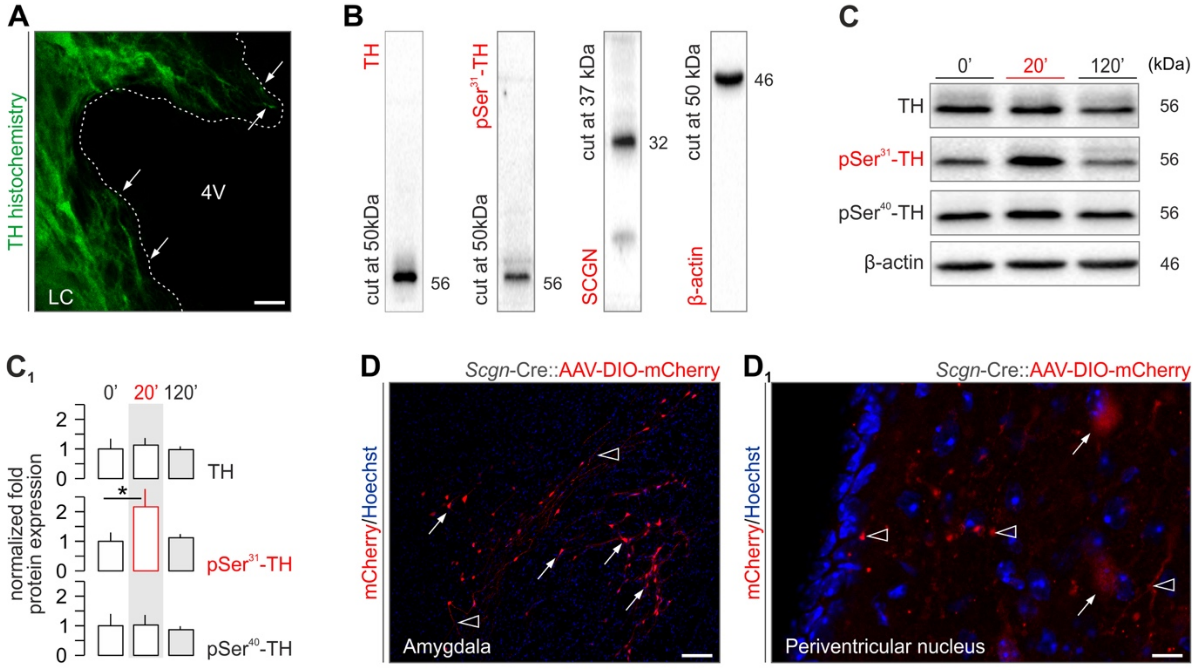

Appendix Figure S2: CNTFRs in LC and experimental models. (related to Figure 3)

A. Tyrosine hydroxylase (TH)$^{+}$ LC neurons contact the ventricular wall (dashed contour) with their fine processes (*arrows*). *Scale bar* = 10 µm.

B. Examples of antibody labeling showing large segments of Western blot membranes (on brain stem homogenates). Note that each antibody produced an immunoreactive band at the predicted size of its target (referred to by numerical labels in kDa).

C. Formalin-induced stress transiently increases TH phosphorylation at Ser^{31} but not Ser^{40} *in vivo*. Representative examples are shown. (**C_1**) Quantitative data from triplicate experiments *$p < 0.05$ at 20 min (Student's *t*-test). Data were expressed as means ± s.e.m.

D. Distribution of mCherry after injecting AAV particles into the amygdala (**D**) and periventricular hypothalamus (**D_1**) of *Scgn*-Cre mice illustrates regional specificity of Cre activity. Ectopic labeling was not observed when comparing mCherry and secretagogin distribution (Mulder *et al*., 2010). *Open arrowheads* pinpoint nerve endings whereas *arrows* show mCherry^{+} perikarya. Data are from *n* = 2 mice/injection site. *Scale bars* = 80 µm (D), 10 µm (D_1).

Appendix Table S1: Demography and use of human subjects.

Case ID	Status	Age (y)	Gender	PMD (h)	Analysis
#125	Control	80	male	4.0	WB
#170	Control	37	male	8.0	WB
#220	Control	63	male	3.5	WB
#228	Control	27	male	8.0	WB
#236	Control	21	male	11.0	WB
#241	Control	81	female	5.0	WB
#281	Control	74	male	2.5	WB
#1	Heart failure	63	male	2.0	WB
#10	Heart failure	42	female	2.0	WB
#11	Heart failure	47	male	2.0	WB
#69	Heart failure	55	male	2.0	WB
#85	Heart failure	68	male	2.5	WB
#233	Heart failure	54	male	2.0	WB
#245	Heart failure	55	male	2.0	WB
#66	Suicide	58	male	4.0	WB
#134	Suicide	31	male	6.0	WB
#138	Suicide	52	male	3.0	WB
#143	Suicide	43	male	3.0	WB
#210	Suicide	39	female	12.0	WB
#235	Suicide	41	male	10.0	WB
#KF1	Control	83	female	7.0	IHC
#KF2	Control	79	male	11.0	IHC

Cases recruited to this study were with the shortest *post-mortem* delay (in hours) allowed legally. “Suicide” includes all forms irrespective of how the act was committed. Age was expressed in years (“y”). Analysis focused on protein detection by Western blotting (WB) or immunohistochemistry (IHC). Case IDs were used to anonymize the subjects included in this study.
