# Supplementary material for: Hypothalamic CNTF volume transmission shapes cortical noradrenergic excitability upon acute stress
Source: EMBO J. 2018 Sep 12;37(21):e100087. doi: 10.15252/embj.2018100087 (PMC6213283; doi:10.15252/embj.2018100087)
Supplement: Supplementary file 2 — Expanded View Figures PDF [file EMBJ-37-e100087-s002.pdf]

Expanded View Figures

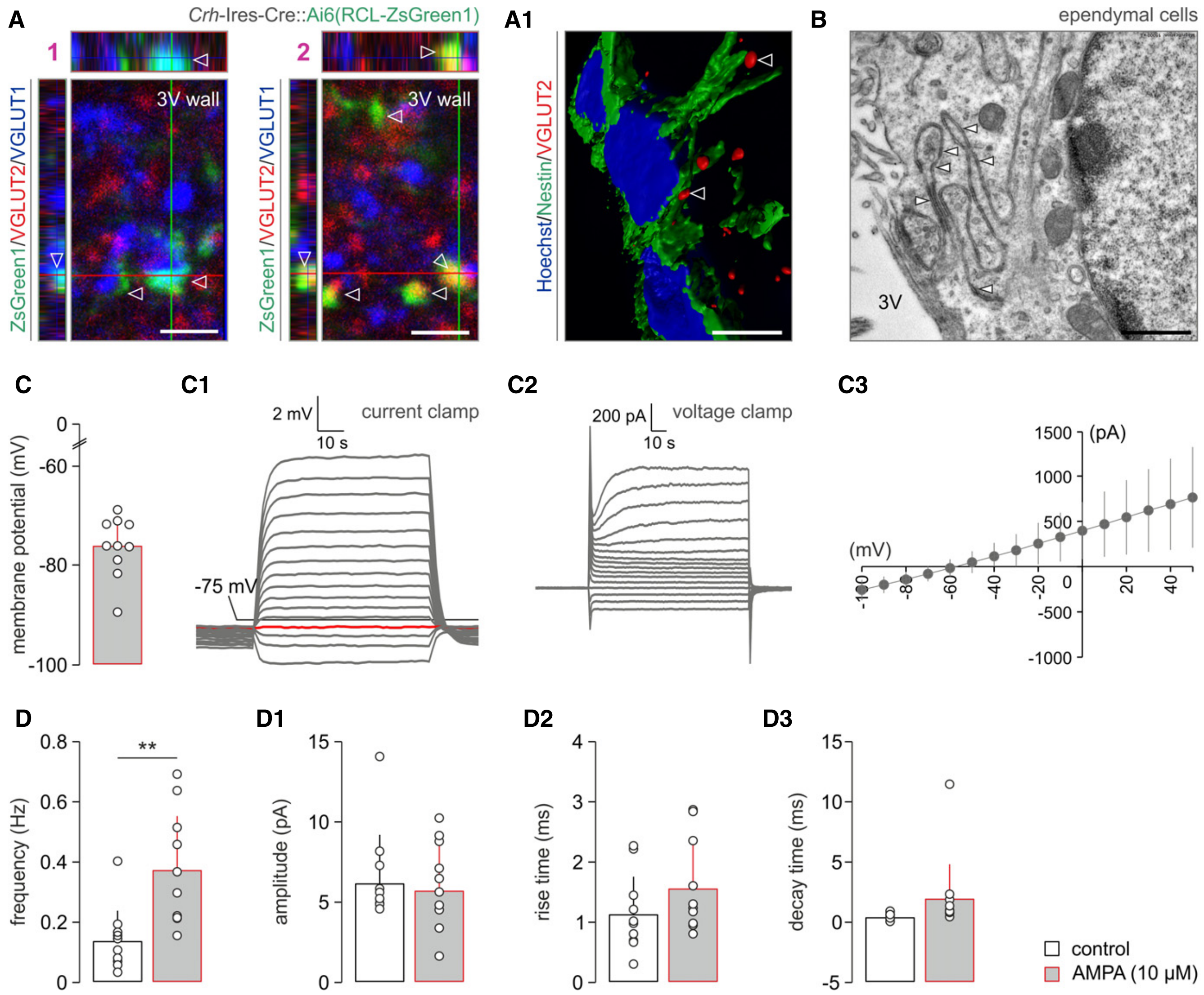

Figure EV1. Glutamatergic inputs to ependymal cells lining the 3rd ventricle (related to Fig 1).

A EGFP+ nerve endings from *Crh-Ires*-Cre::*egfp* mice along the wall of the 3rd ventricle (bottom edge of each image) contained either VGLUT2 (1) or VGLUT1 (2) immunoreactivities (*open arrowheads*). Orthogonal projections. (A1) Three-dimensional rendering of VGLUT2+ nerve endings (*open arrowheads*) along nestin+ ependymal cells. Scale bars = 3 µm (1, 2) and 10 µm (A1).

B Electron micrographs showing gap junctions (*arrowheads*) between the convoluting plasmalemma of ependymal cells. Scale bars = 250 nm.

C Biophysical parameters of ependymal cell membranes, including resting membrane potential (C), and current-clamp (C1) and voltage-clamp (C2) profiles in response to 20 pA and 10 mV depolarization steps, respectively. I-V relationship is shown in (C3). Data from $n = 10$ cells are shown.

D AMPA superfusion significantly increases the frequency of spontaneous postsynaptic currents in ventricular ependyma. $^{**}P < 0.01$ (paired Student's *t*-test), $n = 10$ cells/group. In the meantime, the amplitude (D1), rise time (D2), and decay time (D3) of such currents remained unchanged (for all parameters, $P > 0.1$).

Data information: Data are expressed as means ± s.e.m.

© 2018 The Authors

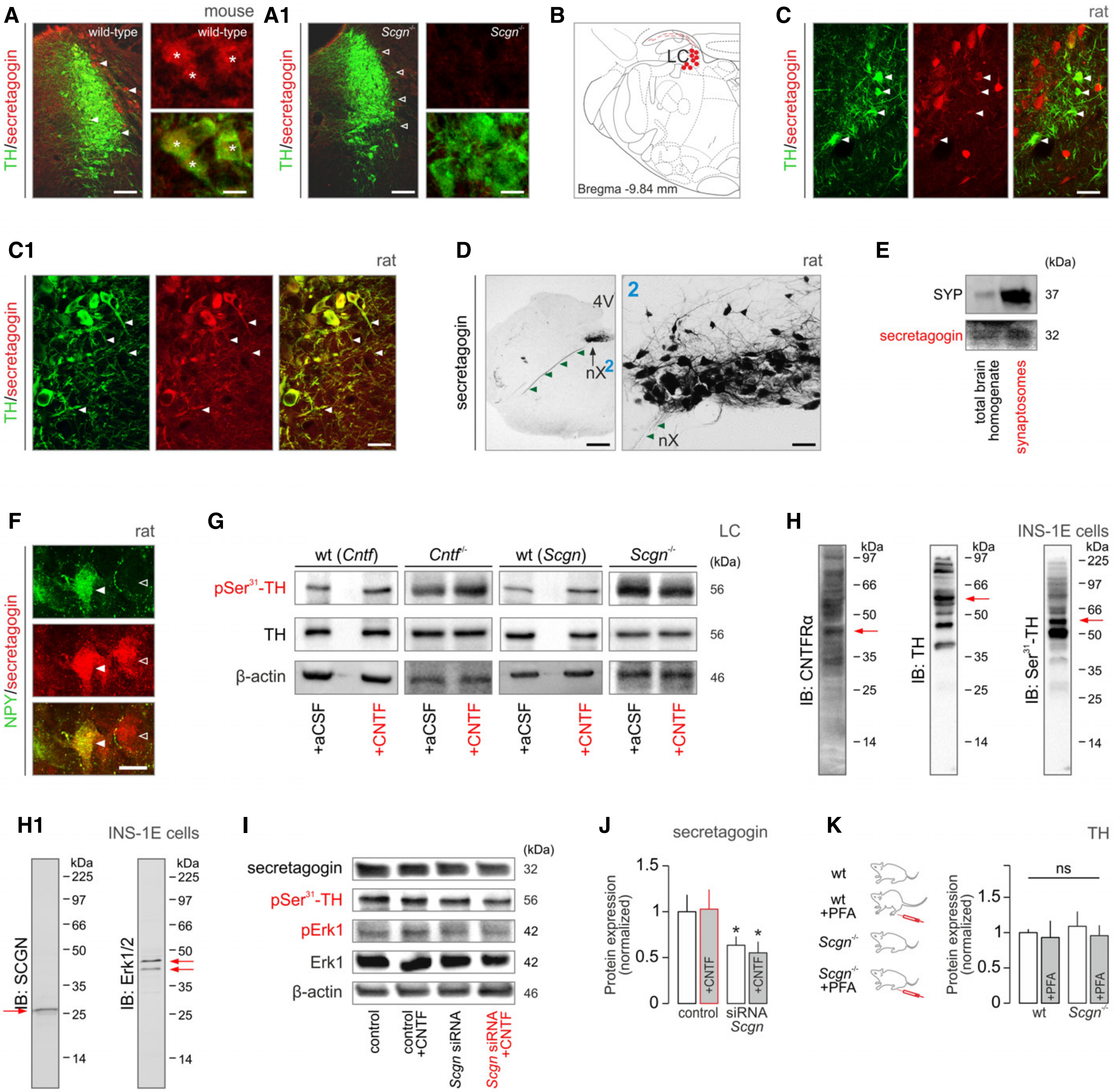
A
mouse
wild-type
wild-type
TH/secretagogin
A1
Scgn-/-
Scgn-/-
B
LC
Bregma -9.84 mm
C
rat
C1
rat
D
rat
secretagogin
4V
nX
2
E
(kDa)
SYP
37
secretagogin
32
total brain homogenate
synaptosomes
F
rat
NPY/secretagogin
G
LC
wt (Cntf)
Cntf-/-
wt (Scgn)
Scgn-/-
(kDa)
pSer31-TH
56
TH
56
β-actin
46
+aCSF
+CNTF
H
INS-1E cells
IB: CNTFRα
IB: TH
IB: Ser31-TH
kDa
H1
INS-1E cells
IB: SCGN
IB: Erk1/2
I
secretagogin
pSer31-TH
pErk1
Erk1
β-actin
control
control +CNTF
Scgn siRNA
Scgn siRNA +CNTF
J
secretagogin
Protein expression (normalized)
control
siRNA Scgn
K
TH
wt
wt +PFA
Scgn-/-
Scgn-/- +PFA
ns

Figure EV2.

Figure EV2. Secretagogin expression in rodent brain stem (related to Fig 4).

A, A1 Labeling specificity of our anti-secretagogin antibody was verified in *Scgn*$^{-/-}$ mice (A1). These experiments also showed that tyrosine hydroxylase (TH)$^{+}$ LC neurons (*) co-express secretagogin in wild-type (*solid arrowheads*) but not null mice (*open arrowheads*). Scale bars = 200 µm (low-power micrographs, *left*) and 10 µm (high-resolution insets, *right*).

B Secretagogin^{+} neurons populate the locus coeruleus (LC) in the rat. Solid red circles pinpoint the general location of perikarya, while fine lines correspond to axons coursing toward the surface (4th ventricle).

C, C1 Secretagogin was expressed in both the somata (*arrowheads*; C) and dendrites (*arrowheads*; C1) of most TH^{+} neurons in the rat, too. Scale bars = 30 µm.

D Secretagogin is localized to axons (*arrowheads*) in the rat. (D) Dorsal nucleus of vagus (nX), "2" shows the nucleus at high resolution. Scale bars = 200 µm (D), 30 µm (2).

E Secretagogin (SCGN) is enriched in the synaptosomal fraction prepared from the mouse prefrontal cortex, lending additional support for its axonal localization. Synaptophysin (SYP) was used to control the enrichment of presynaptic proteins.

F Secretagogin^{+} neurons can co-express neuropeptide Y (NPY) in the LC of the rat. Open and solid *arrowheads* indicate the lack and existence of NPY co-localization in adjacent neurons, respectively. Scale bars = 10 µm.

G Representative Western blots from *Cntf*$^{-/-}$, *Scgn*$^{-/-}$, and wild-type mice with or without intracerebroventricular CNTF treatment.

H, H1 INS-1E cells were used as a cellular model exclusively because of their native co-expression of CNTF receptor-α (CNTFRα), tyrosine hydroxylase (TH), secretagogin (SCGN), and extracellular signal-regulated kinase 1/2 (Erk1/2). Red arrows label target proteins at their predicted sizes. Multiple bands likely represent splice variants, posttranslational modifications, or multimers. Antibodies were also validated histochemically often using knock-out tissues.

I Secretagogin, phospho-Ser^{31}-TH, phospho-Erk1, and total Erk1 expression in INS-1E cells stimulated with CNTF with/without concomitant silencing of secretagogin (*Scgn*) gene expression (for 48 h). Note the lack of Erk1 phosphorylation upon genetic inactivation of secretagogin.

J Significant reduction of secretagogin (SCGN) protein levels upon acute RNAi-mediated gene silencing *in vitro* (for 48 h). CNTF exposure did not affect the efficacy of gene silencing. Experiments were performed in triplicate; *$P < 0.05$ (Student's *t*-test).

K Unchanged tyrosine hydroxylase (TH) expression in wild-type and *Scgn*$^{-/-}$ mice regardless of formalin-induced stress ("+PFA"). These data validate our suggestion on protein phosphorylation, rather than degradation, being a critical step in secretagogin's mechanism of action. ns, non-significant (Student's *t*-test). Data are from $n > 4$ mice/genotype.

Data information: Data are expressed as means ± s.e.m.

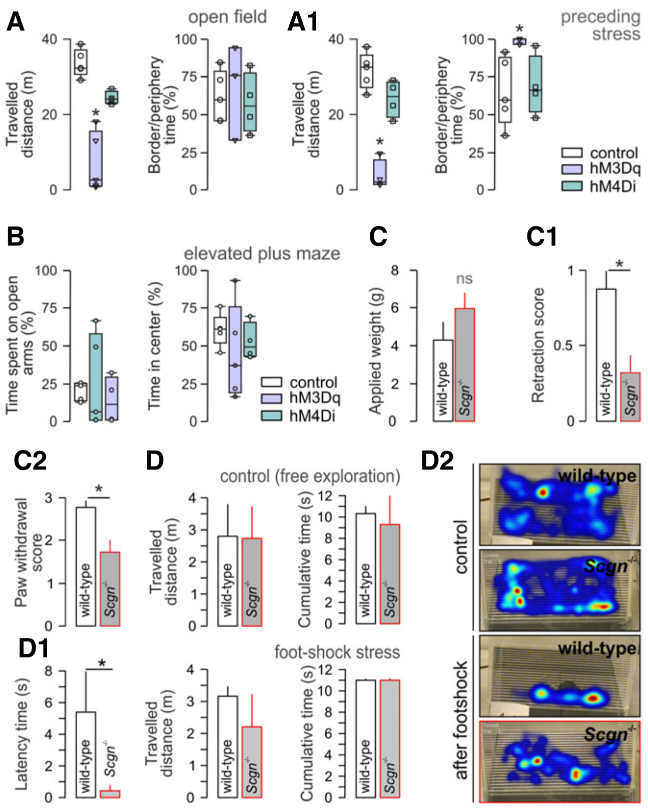

Figure EV3. Behavioral output upon chemogenetic manipulation and in *Scgn*$^{-/-}$ mice (related to Fig 4).

A, A_1 Open-field performance in mice with DREADD constructs expressed in NE neurons with (or without) formalin exposure 20 min prior to behavioral testing. Open labels correspond to values in each individual tested. *$P < 0.05$ vs. control and hM4Di groups.

B Data from elevated plus maze, including time spent on open and in the center of the maze 15–20 min after activating DREADD constructs in NE neurons. Open labels correspond to values in each individual tested.

C *Scgn*$^{-/-}$ mice exhibit normal sensory (pain) thresholds as measured in the von Frey test. (C1, C2) Noxious acute stress in *Scgn*$^{-/-}$ mice vs. wild-type littermates biases sensory behaviors, including the pinna reflex (C1) and paw withdrawal (C2) (n = 5–7/genotype; *$P < 0.05$).

D *Scgn*$^{-/-}$ mice accommodate in a closed environment similar to their wild-type littermates ($P > 0.1$, D). In contrast, *Scgn*$^{-/-}$ mice exhibit only minimal freezing ("latency", D1) in response to repeated stochastic and unavoidable foot-shock. Data represent latency values averaged from five foot-shock sessions. Note that neither the extent of exploratory behavior ("travelled distance") nor the time spent in the center of the closed chamber is different, reinforcing stress resilience rather than accelerated habituation. n = 3 mice/group (*$P < 0.05$ for "latency"). (D2) Cumulative heat-traces of animal movements during representative trials.

Data information: Data are expressed as means ± s.e.m. Behaviors in the open-field and elevated plus maze tests were evaluated by ANOVA using a general linear model with genotype, sex, and treatment being fixed factors followed by appropriate group comparisons. Pairs of treatment groups were analyzed using the Mann–Whitney *U*-test.

© 2018 The Authors

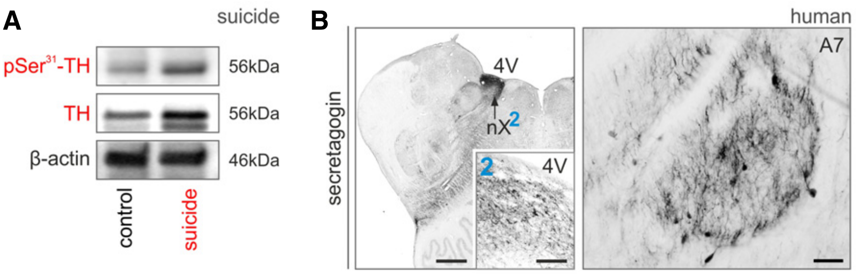

Figure EV4. Secretagogin expression in human brain stem (related to Fig 5).

A pSer[31]-TH and TH expression in suicide individuals. Numerical data are shown in Fig 5B.

B Secretagogin$^+$ neurons densely populate the dorsal nucleus of vagus (nX) and the A7 field in the human brainstem. Scale bars = 1 mm (nX overview, *left*), 150 µm (inset "2"), and 70 µm (A7 group, *right*).

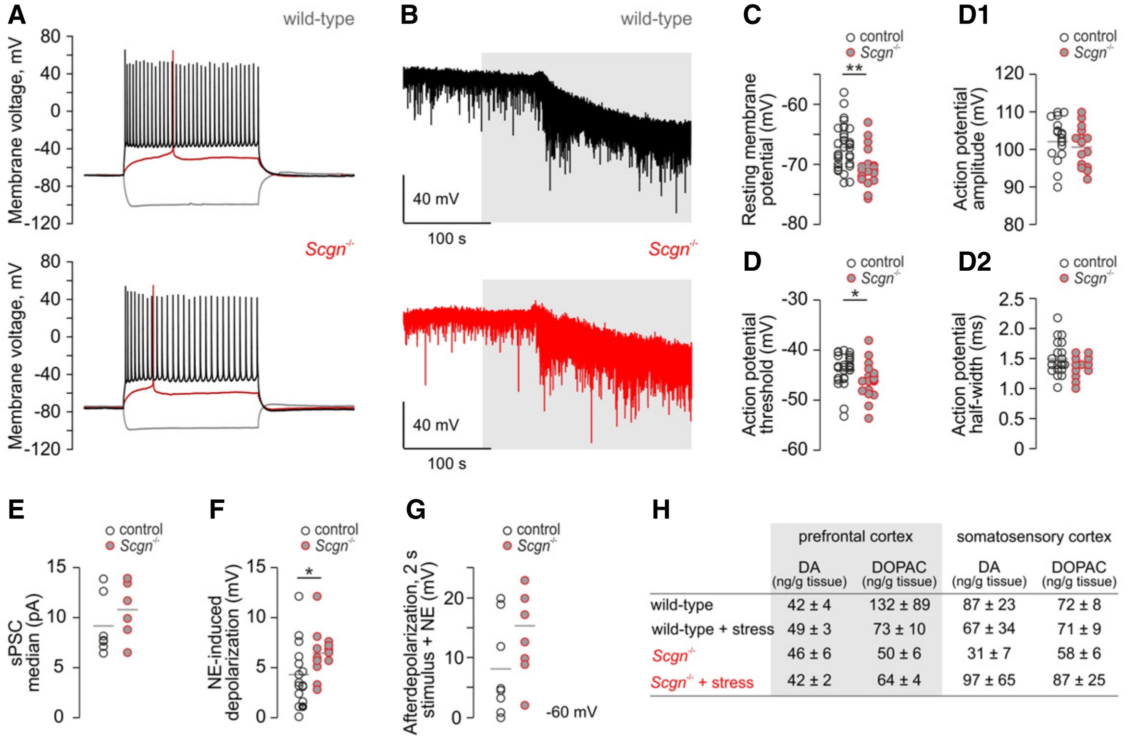

	prefrontal cortex		somatosensory cortex	
	DA (ng/g tissue)	DOPAC (ng/g tissue)	DA (ng/g tissue)	DOPAC (ng/g tissue)
wild-type	42 ± 4	132 ± 89	87 ± 23	72 ± 8
wild-type + stress	49 ± 3	73 ± 10	67 ± 34	71 ± 9
Scgn$^{-/-}$	46 ± 6	50 ± 6	31 ± 7	58 ± 6
Scgn$^{-/-}$ + stress	42 ± 2	64 ± 4	97 ± 65	87 ± 25

Figure EV5. Electrophysiological properties of layer 5 pyramidal cells in the mPFC of *Scgn*$^{-/-}$ vs. wild-type mice (related to Fig 6).

A Representative current-clamp recordings showing significantly hyperpolarized resting membrane potentials of layer (L) 5 pyramidal cells in the mPFC of *Scgn*$^{-/-}$ mice immediately upon membrane rupture (see panel C for quantitative data).

B Spontaneous postsynaptic currents in *Scgn*$^{-/-}$ mice. Neurons were clamped at positive voltages to preferentially resolve excitatory postsynaptic currents. Note the markedly increased frequency of postsynaptic excitation in *Scgn*$^{-/-}$ mice.

C Quantitative demonstration of a negative shift in neuronal resting membrane potential in *Scgn*$^{-/-}$ pyramidal cells; $^{**}P < 0.01$, $n = 33$ (control) vs. $n = 18$ (*Scgn*$^{-/-}$).

D Action potential parameters for L5 pyramidal cells in *Scgn*$^{-/-}$ mice vs. wild-type littermates. (D) While action potential threshold was significantly reduced, neither its amplitude (D1) nor its half-width changed in *Scgn*$^{-/-}$ cells (D2). $^{*}P < 0.05$, $n = 23$ (control) vs. $n = 16$ (*Scgn*$^{-/-}$).

E Quantitative depiction of spontaneous postsynaptic currents (sPSCs) isolated as in panel (B). Note a tendency toward an increase in sPSC frequency in *Scgn*$^{-/-}$ cells. Each data point represents the median value (pA) of recording sweeps per cell. $n = 8$ (control) vs. $n = 7$ (*Scgn*$^{-/-}$).

F Membrane depolarization upon superfusion with 10 µM norepinephrine. On average, *Scgn*$^{-/-}$ neurons produced larger responses than their wild-type counterparts. $^{*}P < 0.05$, $n = 15$ (control) vs. $n = 13$ (*Scgn*$^{-/-}$).

G Afterdepolarization evoked by 2-s stimulus in the presence of NE with each neuron pre-clamped to −60 mV. $n = 8$ (control) vs. $n = 7$ (*Scgn*$^{-/-}$).

H Tissue levels of dopamine (DA) and 3,4-dihydroxyphenylacetic acid (DOPAC), its major metabolite, in the medial prefrontal and somatosensory cortices of wild-type and *Scgn*$^{-/-}$ mice that optionally underwent acute formalin stress. The stress-induced increase in tissue norepinephrine (NE) content in wild-type but not *Scgn*$^{-/-}$ mice is shown in Fig 6E. Data are from $n > 4$ animals/group.

Data information: Results in panels (C, D, and F) were statistically analyzed by one-way ANOVA.

© 2018 The Authors
