# Supplementary material for: Hypothalamic CNTF volume transmission shapes cortical noradrenergic excitability upon acute stress
Source: EMBO J. 2018 Sep 12;37(21):e100087. doi: 10.15252/embj.2018100087 (PMC6213283; doi:10.15252/embj.2018100087)
Supplement: Supplementary file 3 — Movie EV1 [file EMBJ-37-e100087-s003.zip › MovieEV1/EMBOJ-2018-MovieEV1Legend.docx]

Annex (Supplementary Material) to:

Hypothalamic CNTF volume transmission shapes cortical noradrenergic excitability upon acute stress (A. Alpár *et al*., The EMBO Journal)

**Table of Contents**

**Movie EV1:** Open field behavior of control mouse.

**Appendix Videos S1: Open field behavior of control mouse.**

Open-field behavior of a control mouse.
