# Supplementary material for: Hypothalamic CNTF volume transmission shapes cortical noradrenergic excitability upon acute stress
Source: EMBO J. 2018 Sep 12;37(21):e100087. doi: 10.15252/embj.2018100087 (PMC6213283; doi:10.15252/embj.2018100087)
Supplement: Supplementary file 4 — Movie EV2 [file EMBJ-37-e100087-s004.zip › MovieEV2/EMBOJ-2018-100087R2_MovieEV2.docx]

Annex (Supplementary Material) to:

Hypothalamic CNTF volume transmission shapes cortical noradrenergic excitability upon acute stress (A. Alpár *et al*., The EMBO Journal)

**Table of Contents**

**Movie EV2:** Open field behavior of hM3Gq DREADD mouse.

**Appendix Videos S2: Open field behavior of hM3Gq DREADD mouse.**

Open-field behavior of *Scgn*-Cre mice treated with activating DREADD (hM3Gq; viral delivery in LC) 17 days prior to probing with CNO (2mg/kg of body weight). CNO was administered 15-20 min before video tracking.
