# Supplementary material for: Hypothalamic CNTF volume transmission shapes cortical noradrenergic excitability upon acute stress
Source: EMBO J. 2018 Sep 12;37(21):e100087. doi: 10.15252/embj.2018100087 (PMC6213283; doi:10.15252/embj.2018100087)
Supplement: Supplementary file 5 — Movie EV3 [file EMBJ-37-e100087-s005.zip › MovieEV3/EMBOJ-2018-100087R2_MovieEV3Legend.docx]

Annex (Supplementary Material) to:

Hypothalamic CNTF volume transmission shapes cortical noradrenergic excitability upon acute stress (A. Alpár *et al*., The EMBO Journal)

**Table of Contents**

**Movie EV3:** Open field behavior of hM4Gi DREADD mouse.

**Appendix Videos S3: Open field behavior of hM4Gi DREADD mouse.**

Open-field behavior of *Scgn*-Cre mice treated with activating inactivating DREADD (hM4Gi; viral delivery in LC) 17 days prior to probing with CNO (2mg/kg of body weight). CNO was administered 15-20 min before video tracking.
